# A versatile, multi-laser twin-microscope system for light-sheet imaging

**DOI:** 10.1063/1.5144487

**Published:** 2020-05-27

**Authors:** Kevin Keomanee-Dizon, Scott E. Fraser, Thai V. Truong

**Affiliations:** 1 Translational Imaging Center, Dornsife College of Letters, Arts and Sciences, and Viterbi School of Engineering, University of Southern California, Los Angeles, California 90089, USA; 2 Translational Imaging Center, Molecular and Computational Biology Section, Department of Biological Sciences, University of Southern California, Los Angeles, California 90089, USA

## Abstract

Light-sheet microscopy offers faster imaging and reduced phototoxicity in comparison to conventional point-scanning microscopy, making it a preferred technique for imaging biological dynamics for durations of hours or days. Such extended imaging sessions pose a challenge, as it reduces the number of specimens that can be imaged in a given day. Here, we present a versatile light-sheet imaging instrument that combines two independently controlled microscope-twins, built so that they can share an ultrafast near-infrared laser and a bank of continuous-wave visible lasers, increasing the throughput and decreasing the cost. To permit a wide variety of specimens to be imaged, each microscope-twin provides flexible imaging parameters, including (i) operation in one-photon and/or two-photon excitation modes, (ii) delivery of one to three light-sheets via a trio of orthogonal excitation arms, (iii) sub-micron to micron imaging resolution, (iv) multicolor compatibility, and (v) upright (with provision for inverted) detection geometry. We offer a detailed description of the twin-microscope design to aid instrument builders who wish to construct and use similar systems. We demonstrate the instrument’s versatility for biological investigation by performing fast imaging of the beating heart in an intact zebrafish embryo, deep imaging of thick patient-derived tumor organoids, and gentle whole-brain imaging of neural activity in behaving larval zebrafish.

## I. INTRODUCTION

Most of what we recognize as the phenomena of life are not properties of stationary structures but emerge from dynamic interactions among many elements over time. Modern optical microscopy methods offer an efficient means for non-invasive, high-resolution observation of many of life’s most fascinating phenomena.^30^ The difficulty is that light imaging involves unavoidable tradeoffs between spatial resolution, acquisition speed, field-of-view, penetration depth, and the limited photon budget from the sample.^1^ Considerations of the photon budget are crucial to biological imaging, as there are a finite number of photons that a given fluorophore can emit before it is bleached, and high light doses on the specimen can lead to photo-induced toxicity. It is thus critical that the excitation light be used as efficiently as possible and the emitted photons be collected as efficiently as possible while striking the optimal imaging compromises for whatever the application demands.

Over the past decade, there have been a series of important developments in light-sheet microscopy, a century-old technique^2^ (also known as selective-plane illumination microscopy; SPIM).^3^ SPIM decouples the illumination and detection paths by using separate optics to excite and detect fluorescence: a cylindrical illumination lens is used to project a static, thin, two-dimensional (2D) sheet of light coincident to the focal plane of a detection objective lens. In contrast to confocal laser scanning microscopy and other point-scanning techniques that acquire volumetric information one voxel at a time, light-sheet excitation permits an entire 2D plane of fluorophores to be excited and detected with high signal-to-noise ratio, high imaging speed, and minimal light exposure to the sample. Developments in light-sheet microscopy techniques^4–11^ have led to cutting-edge applications across a range of fields^4–7,10,12,13^ from developmental biology^5,14^ to neuroscience.^10,12,15–17^ Each of these modifications of light-sheet imaging has involved their own tradeoffs in performance, complexity and expense of the microscope optics, and expenditure of the photon budget. Comprehensive reviews of light-sheet development can be found elsewhere (see Ref. 5)—we highlight below several key developments that address the experimental demands that motivate the development of our instrument.

**FIG. 1.**
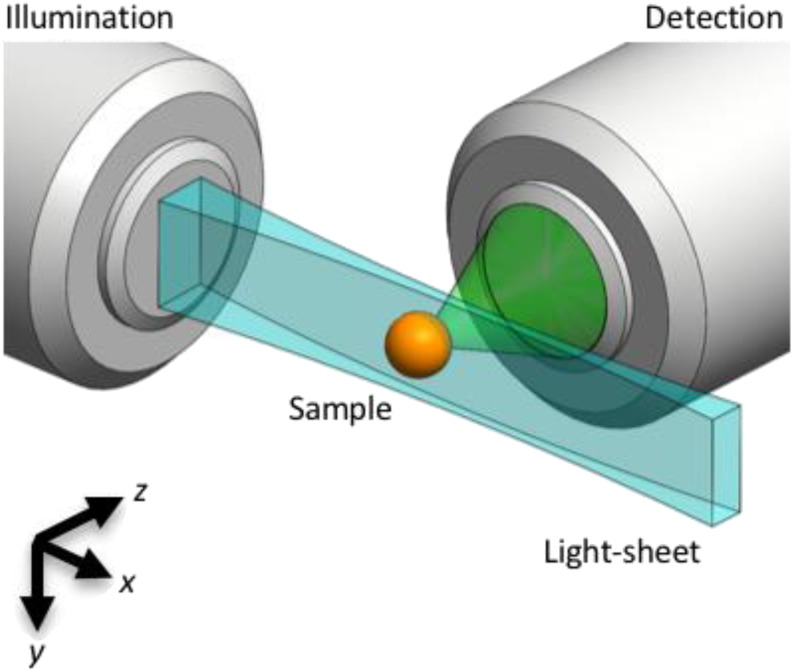
Light-sheet microscopy principle. A light-sheet (blue) can be created by dynamically scanning, along the y direction, a focused Gaussian beam that propagates in the *x* direction. The focusing is achieved via a low numerical aperture illumination lens. The fluorescence generated by the illuminated (*x–y*) plane is imaged (green) by an orthogonally positioned wide-field microscope. Axial (*x*–*y*) sections of the 3D sample are captured either by scanning the sample (orange) through the stationary focal plane or by scanning the light sheet and detection focal plane through the stationary sample.

A key development was to create light-sheets by dynamically scanning a focused Gaussian beam, generated via a low-numerical-aperture (NA) lens, across the plane (Fig. 1).^18^ This scanned Gaussian-beam light-sheet approach (also termed digital scanned laser light-sheet fluorescence microscopy; DSLM)^18^ provides better spatial illumination uniformity, higher light throughput, and more precise spatial control over the selected plane of interest compared to a static 2D light-sheet at the cost of replacing the simple cylindrical lens with the expense and complexity of a scanning galvanometer (galvo) mirror and associated optics and electronics. This task has been simplified by the commercial availability of integrated electro-optical galvo scanning modules.

The penetration depth of one-photon excitation light-sheet microscopy (1P-SPIM) was improved in two-photon light-sheet microscopy (2P-SPIM), where nonlinear excitation was added to the DSLM implementation.^19^ 2P-SPIM has proven successful in relatively thick or optically dense samples, imaging up to twofold deeper than DSLM/1P-SPIM, and more than tenfold faster and 100-fold lower peak intensity than conventional 2P point-scanning microscopy. In addition, the near-infrared (NIR) light used for 2P excitation is invisible to many animals, which avoids unintended visual stimulation.^20^ The disadvantages of 2P excitation are the extra laser cost, lower fluorophore absorption cross section (leading to lower maximum imaging speed), and less amenability to multicolor imaging compared to 1P excitation.^5,9^ Thus, for the best flexibility to handle a wide variety of samples, both 1P and 2P excitation modes are desirable. Because both use a low-NA illumination lens to generate an axially extended Gaussian focus, they can be conveniently carried out on the same light-sheet instrument.^19,21^

A major expense of SPIM imaging setups is the laser source used for excitation, especially given that multiple lasers are used for multicolor imaging. For 2P-SPIM the ultrafast laser can more than double the equipment cost, which has limited its adoption, despite its superior performance in optically challenging samples. Even for well-funded laboratories, laser sources tied to a single microscope are not cost-effective. Since there is an upper limit to the amount of laser power that can be delivered to any specimen without perturbing it, most implementations waste well more than half of the total laser power available.

Another practical challenge comes from the often dual need of continuously imaging to generate time-lapses and of imaging a large number of samples to obtain statistically significant results. While the low photodamage of SPIM allows biological processes to be imaged for a duration of several hours or days, a tradeoff exists between such prolonged imaging sessions and the number of samples that can be imaged in a given day using a single instrument.

Here, we describe the flex-SPIM, which combines two independently controlled light-sheet microscope-twins that share an ultrafast NIR laser and a bank of continuous-wave (CW) visible lasers. This permits two specimens to be imaged simultaneously for far less than the cost of two multi-laser microscopes. Each microscope-twin has built-in modularity for tailoring its use on diverse samples and scientific questions. In Sec. II, we describe the flex-SPIM design in detail for those who wish to construct and use a similar instrument. In Sec. III, we test the performance against our design objectives by imaging three challenging specimens: the beating larval zebrafish heart, patient-derived tumor organoids, and whole-brain neural dynamics in behaving zebrafish. The results demonstrate the ease of adapting the flex-SPIM for application-specific light-sheet imaging.

## II. INSTRUMENT DESIGN, INTEGRATION, AND CONFIGURATION

**FIG. 2.**
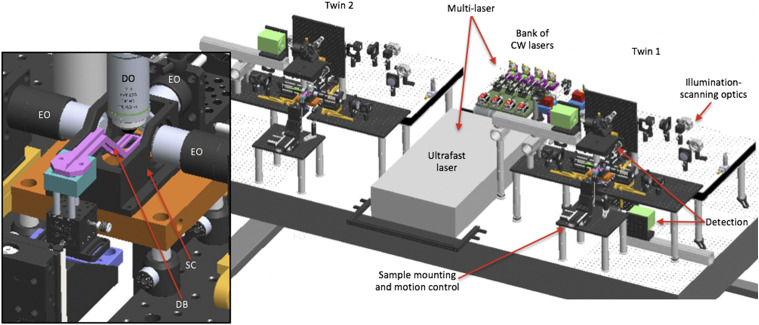
3D opto-mechanical model of the twin-microscope system mounted on a 5 × 10 ft^2^, anti-vibration optical table. Model shows the multi-laser subsystem shared between microscope-twin-1 (right) and microscope-twin-2 (left). Twin-1 has the four functional subsystems labeled and features an implementation of both upright and inverted detection. Brief descriptions of each functional subsystem are provided in Table I. The inset shows a detailed view of the sample chamber (SC), the dive bar (DB) used to hold the sample, three excitation objectives (EO) to deliver excitation light-sheets to the sample, and the detection objective (DO) to collect emitted fluorescence from the sample.

The flex-SPIM draws on lessons learned from proof-of-principle studies^19,21^ with a new imaging technology to meaningful scientific results;^22–24^ several years of interactions with end users at advanced imaging centers (at the California Institute of Technology and the University of Southern California), the 2P-SPIM inventors, and other instrument builders; and thus integrates the following combination of improvements:

• Two independent microscope-twins share the same multi-laser source (Fig. 2), dramatically reducing instrument cost—more than 30% savings. This also minimizes the on-going maintenance and operation costs, since lasers need service and/or replacement after a finite lifetime.
• The twin architecture doubles the 1P- and 2P-SPIM imaging throughput and increases the variety of specimens or imaging modes, when compared to a single microscope.
• The system is “flexible” by design, with an opto-mechanical configuration that is both open and modular, providing a straightforward path to instrument evolution and customization for different samples and applications. Three orthogonal illumination arms offer easy matching to different specimens, enhancing illumination uniformity or increasing optical coverage for larger and more opaque samples.^25^ Switching from high lateral spatial resolution (sub-micrometer) to a lower resolution (∼micrometer) with a larger field-of-view requires only a simple adjustment to the detection subsystem. Each twin can further be configured in upright and/or inverted detection geometries to accommodate a diversity of specimens (Fig. 2).

**FIG. 3.**
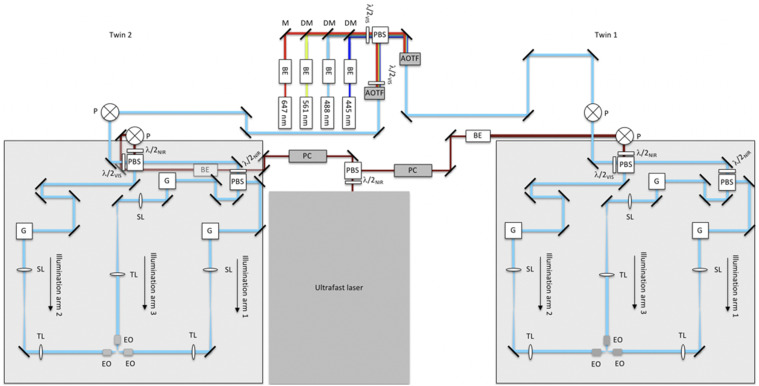
Schematic diagram of multi-laser and illumination-scanning optics subsystems of the instrument. Visible light from the continuous-wave laser bank is fed into microscope-twin 1 (right) and microscope-twin 2 (left) via polarization beamsplitting optics [consisting of a polarizing beamsplitter (PBS) and half-wave plate]. Acousto-optic tunable filters (AOTFs) are used to select the visible wavelengths and adjust the power independently for each twin. The near-infrared (NIR) light from the ultrafast laser is routed similarly using Pockels cells (PCs) to adjust the NIR power independently for each twin. The visible and NIR beams are raised onto 24 × 36 in.^2^ optical breadboards by using periscopes (P). Polarization beamsplitting optics are used both to combine the visible and NIR beams and to split the combined beam into two paths (illumination arms 1 and 2). Illumination arm 1 is further split into two paths through polarization beamsplitting optics, creating a total of three illumination arms. Each illumination arm directs light to the sample through the excitation objectives (EO). BE—beam expander, M—mirror, DM—dichroic mirror, λ/2—half-wave plate, where the subscripts VIS and NIR refer to the visible and near-infrared wavelengths, respectively, G—2D scanning galvo mirrors, SL—scan lens, and TL—tube lens. BE in the NIR twin-2 path appears gray because it is underneath the optical breadboard.

The flex-SPIM consists of four functional subsystems (Fig. 2) and two modules (Table I) and sits on a 5 × 10 ft^2^, anti-vibration optical table (Fig. S1). The schematic diagram of the integrated illumination paths is shown in Fig. 3; the corresponding 3D opto-mechanical model is shown in Fig. 2 and the complete computer-aided design (CAD) is available upon request. Whenever possible, commercially available hardware components are used; however, both basic machining of off-the-shelf parts and the fabrication of custom components are required (Table SI). Most standard optical elements are mounted in Thorlabs 30 mm or 60 mm cage components. Beam steering mirrors shared by both the ultrafast and CW lasers (illumination-scanning optics) have protected silver coatings, whereas those used by the ultrafast or CW lasers alone have broadband dielectric coatings.

**TABLE I.** Flex-SPIM functional subsystems and modules. CW—continuous-wave, polarization beamsplitting optics—half-wave plate and polarizing beamsplitter, AOTF—acousto-optic tunable filter, galvo—galvanometer, and LED—light-emitting diode.

Subsystem/module	Description	Main components^a^
Multi-laser	Laser modulation and beam routing	CW lasers, beam expander, dichroic mirrors, polarization beamsplitting optics, AOTFs, and mirrors Ultrafast laser, polarization beamsplitting optics, beam expander, Pockels cells, and mirrors
Illumination-scanning optics	Generating the light-sheet	Scanning galvo mirrors and optics, mirrors, and objectives
Detection	Image capture	Scientific camera, tube lens, filter wheel, filters, and objective
Sample mounting and motion control	Holding and imaging the 3D sample	Custom sample chamber/holder, motion stages, and piezoelectric z-stage
Instrument control	Control and timing of components	Acquisition computer, national instruments PXI, system mainframe and scaling amplifiers
Auxiliary	Illumination masks and view-finding	Camera, LED, filter, and aluminum masks

^a^ The main parts for each subsystem and module used to build the flex-SPIM are listed in Table SI.

### A. Multi-laser subsystem

CW visible light used for single- and multi-color imaging via linear excitation is provided by a bank of CW lasers (445 nm, 488 nm, 561 nm, and 647 nm), collimated and expanded to a 1/e^2^ diameter of 1.5 mm, and combined into a co-linear beam using broadband and dichroic mirrors (see Table SI).^26^ The combined beam is then split into two paths of equal length and power through polarization beamsplitting optics [consisting of a half-wave plate mounted on a rotation mount and polarizing beamsplitter (PBS)], delivering light to each microscope-twin. Each light path passes through acousto-optic tunable filters (AOTFs), which are used to select the wavelengths and adjust the power independently for each twin. The AOTFs (Table SI) require the input laser beam polarization to be linearly orthogonal to the baseplate (*s*-polarization) to maximize the diffraction efficiency and ensure chromatic co-linearity of the modulated beam. Alternatively, the input beam can be *p*-polarized if the AOTF crystal output face is used as the “input” face due to the Helmholtz reciprocity principle.^27^ Because of the upstream polarizing beamsplitter used, the beams are *p*-polarized for the twin-1 path and *s*-polarized for the twin-2 path. As such, the AOTF in the twin-1 path is mounted so that the *p*-polarized beam enters the AOTF through its output face; the AOTF in the twin-2 path is mounted conventionally. A half-wave plate can be placed in front of the AOTF to fine-tune the polarization direction of the beam entering the AOTF and thereby maximize the diffraction efficiency by the AOTF when more excitation energy is required at the sample, as we have implemented in the twin-2 path (Fig. 3). Note that alternatively, similar performance could be achieved by placing a half-wave plate in front of the AOTF in the twin-1 path to rotate the beam’s polarization so that it enters the AOTF conventionally.

The tunable NIR ultrafast mode-locked laser used for single- and multi-color imaging via nonlinear excitation is split into two paths of equal length and power through polarization beamsplitting optics, delivering NIR light to both microscope-twins. Note that the linear polarizations of the beams for the twin-1 path and the twin-2 path are orthogonal to each other. Each path passes through a Pockels cell to control the power independently for each twin (Fig. 3). Each Pockels cell is rotated to match its input polarization requirement and hence maximize its extinction ratio, removing the need for additional half-wave plates and power losses from their imperfections. Following the Pockels for each path, the beams are expanded to a 1/e^2^ diameter of 2.2 mm. A long-pass filter (800-nm cutoff) is mounted upstream of the beam expander to block any undesired residual wavelengths from the ultrafast laser source.

### B. Illumination-scanning optics subsystem

Visible and NIR beams from the multi-laser subsystem are each raised onto a pair of 24 × 36 in.^2^ optical breadboards, one for each twin, by periscopes (Fig. 3). Polarizing beamsplitters are used to both combine the visible and NIR beams into a co-linear beam and to split the combined beam into two paths (illumination arms 1 and 2). Visible and NIR half-wave plates, each mounted in manual rotation mounts, are used to adjust the laser power (splitting ratio) delivered to illumination arms 1 and 2. Illumination arm 1 is further split into two paths through polarization beamsplitting optics, creating a total of three illumination paths. Rotation of the NIR half-wave plates before the polarizing beamsplitter can be used to adjust the relative laser power into illumination arms 1 and 3 (Fig. 3). The path lengths of all three illumination arms are routed so that they are equal.

The beams from each illumination arm are sent to a 2D scanning galvo mirror positioning system. The first galvo mirror rapidly scans the beam laterally to synthesize the light-sheet (in the *x*–*y* plane), and the second galvo mirror, which is conjugate to the back pupil of the excitation objective lens, translates the virtual light-sheet along the (*z*) detection axis. Following the scanning system, each illumination beam passes through a scan lens (achromatic doublet; see the supplementary material Note 1); a tube lens, and a low magnification, low-NA, long-working-distance excitation objective lens. The distances between pairs of lenses form a 4*f* arrangement (the distance between pairs of lenses are equal to the sum of their focal lengths).

Three excitation objective lenses, mounted orthogonally to each other, direct the illumination light toward the sample (Fig. 3). Depending on the sample properties, any combination of the excitation objectives can be used, either sequentially or simultaneously. Small and/or transparent samples, for example, may benefit from single-sided illumination with 1P excitation, whereas relatively large and thick samples may benefit from the uniform illumination coverage offered by using all three objectives with 2P excitation.

The illumination NA for 1P and 2P is ∼0.02 and 0.03, respectively, yielding fluorescence Gaussian-beam light-sheets of ∼10 *µ*m in average thickness across an extent of ∼400 *µ*m, as shown in Fig. 7(d). Note that to produce similar nominal fluorescence light-sheet thicknesses and extents, different NAs are needed for 1P vs 2P due to the difference in laser wavelength and the linear (1P) vs quadratic (2P) dependence of the fluorescence on the laser intensity.^19^ Scanning of the first galvo yields an effective (*x*–*y*) field-of-view of ∼400 × 1000 *µ*m^2^. The chosen light sheet thickness ensures that we are able to resolve single neurons (6–8 *µ*m in size) throughout the entire ∼400 × 800 × 250 (*x*–*y*–*z*) *μ*m^3^ brain of zebrafish larva at 5 days-post-fertilization (dpf).^20,28^

Light throughput for each twin, defined as the total measured laser power at the sample from the three illumination arms divided by 50% of the measured power at the laser output (for 1:1 split into each twin) for the ultrafast laser (taken at λ = 900 nm) is ∼60%. Light throughput in the visible regime, taken by throughput measurements across each CW laser line is ∼6%. The lower visible light throughput is expected since most of the illumination-scanning optics were selected to optimize NIR throughput and maximize 2P excitation efficiency (Table SI). Both the ultrafast and CW laser sources are able to simultaneously run experiments on both twins with independent power control.

### C. Detection subsystem

The sheet of fluorescence signal generated at the sample is collected by an orthogonally positioned water-immersion detection objective lens (20×, 1.0 NA; see Table SI), mounted to a piezoelectric (piezo) collar. The high-NA objective, with low intrinsic magnification, not only enables high-resolution imaging across large volumes but also importantly maximizes light-collection, which is critical for maintaining acceptable signal-to-noise while minimizing the excitation laser power to reduce photodamage to live samples. The fluorescence signal passes through a filter wheel equipped with emission filters to block the excitation light and transmit the fluorescence signal emitted by the sample; the emission filters are optimized for the transmission of common fluorophores spanning the visible spectrum (Table SI). A tube lens forms the primary image of the fluorescence signal onto a scientific complementary metal-oxide-semiconductor (sCMOS) camera.

**FIG. 4.**
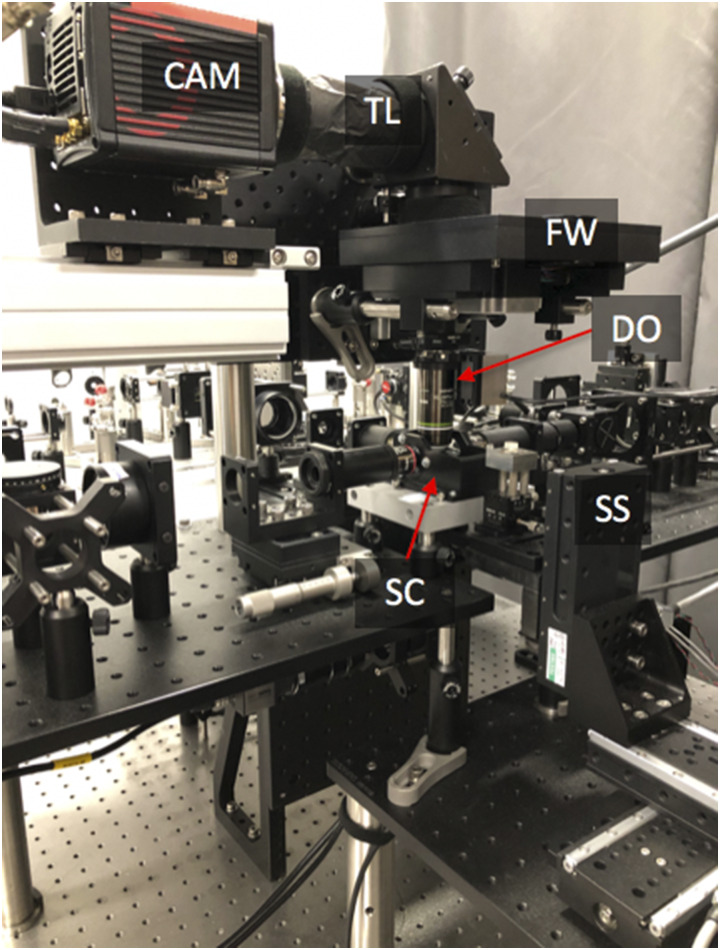
Photograph of an assembled microscope-twin with upright detection. SC—sample chamber, SS—3D stage stack-up, DO—detection objective, FW—filter wheel, TL—tube lens, and CAM—camera.

Different magnifications are achieved with the same detection objective by using tube lenses of different focal lengths, with the rail-mounted fluorescence camera providing the necessary distance adjustments (Figs. 2 and 4). Generally, tube lenses need to have a large enough diameter to capture the detection optical aperture and good performance to minimize chromatic and geometric aberrations.^30^ Lower or higher magnification requires lenses with shorter or longer focal lengths, potentially exacerbating aberrations or making the instrument footprint larger, respectively. We find that the practical range of magnification is between 44× and 11× for our instrument (Table SI), given the available off-the-shelf double achromats (used as tube lenses), the size constraint of standard opto-mechanical components, and the desire to keep our instrument reasonably compact.

Our detection subsystem design choices described above represent a balanced compromise to achieve an economical light-sheet instrument with adjustable magnification, maximizing resolution while maintaining a large field-of-view. As will be shown later, the instrument provides cellular to sub-cellular resolution, sufficient for a wide range of applications. We note that the maximum diffraction-limited resolution, as determined theoretically by the NA of the detection objective, is not expected to be achieved, given the practical design choices that we have made. First, at the magnifications used, the camera pixel size is not small enough to provide diffraction-limited spatial sampling according to the Nyquist criterion.^30^ Second, independent of the magnification used, we do not expect our detection path to be fully aberration-free due to our choice of using economical off-the-shelf double-achromats as tube lenses, rather than more expensive specialized tube lenses (which often only exist with limited focal lengths). If higher resolution is desired with our system, a detection objective with higher NA and/or higher intrinsic magnification could be used in combination with the matched tube lens from the same manufacturer.

Depending on the sample properties, the detection subsystem can be arranged for upright and/or inverted configurations. We have experimentally implemented an upright configuration (Fig. 4), as it is the most optimized for the biological samples presented here. We implemented an inverted configuration in the CAD model shown in Fig. 2, where the same detection subsystem was designed to be mounted in either an upright or inverted configuration. Owing to the system’s arrangement of opto-mechanical components (Figs. 2–4), switching between detection geometries or changing the overall magnification is relatively straightforward.

### D. Sample mounting and motion control subsystem

The sample chamber has three side windows for the excitation objectives as well as a bottom window to provide an additional view of the specimen. The sample chamber is open at the top and is filled with imaging buffer; the open-top allows the detection objective to be liquid-immersed and the sample holder to be inserted. The sample chamber sits on a custom heat exchanger that has circulatory channels for temperature regulated fluid flow, which can be used to keep the medium-filled sample chamber at a specific temperature.

**FIG. 5.**
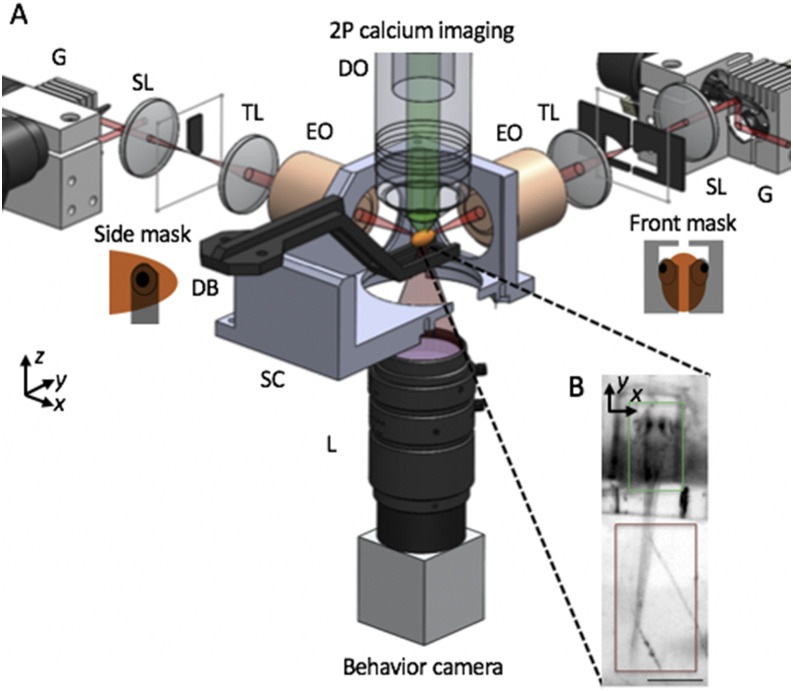
SPIM imaging of intracellular calcium for capturing neuronal activity. (a) Schematic of apparatus for imaging of neural activity during various behaviors in the larval zebrafish. Sheets of laser light are synthesized by quickly scanning the pulsed illumination beam (red) with galvo mirrors (G). 2P light-sheets are delivered to the agarose-embedded head of the animal with excitation objectives (EO) from the side and front arms. The side masks cover each eye on the sides of a horizontally oriented zebrafish, while the front mask covers both eyes, enabling access to neurons between the eyes. 2P-excited calcium fluorescence signal is collected through an upright detection objective (DO) and onto a scientific CMOS camera. A triggerable wide-field camera is positioned below the sample chamber (SC) to provide a wide-field, low-resolution view of the sample, as shown in (b). During a typical neural imaging experiment, the zebrafish larva is mounted in a caddy, which in turn is mounted to the dive bar (DB) underneath the DO. Within the caddy, the zebrafish’s head is immobilized in agarose, while the tail is free, permitting the monitoring of zebrafish behavior through tail movement. SL—scan lens, TL—tube lens, SC—sample chamber, and L—camera lens. The third illumination arm, emission filter, detection TL, scientific camera, light-emitting diode, and filter for behavior channel are not shown. Insets in (b) highlight that the calcium fluorescence channel (green) is recorded from the zebrafish brain, while the behavioral channel (dark red) monitors the tail movement of the animal. Scale bar: (b) 400 *µ*m.

The sample holder is comprised of two parts: (i) a caddy that holds the specimen and (ii) a “dive bar” that holds the caddy and connects it to the stack-up of motion-control stages. Caddies can be used for agarose embedding of the sample or adapted to specific applications. For example, the caddy for imaging neural activity in behaving zebrafish [Fig. 5(b)] immobilizes the specimen’s head with 1.5% low-melting agarose gel (to record cellular resolution whole-brain neural activity), leaving the tail free to move (to record swimming behavior). With one end of the dive bar holding the caddy immersed in the imaging buffer, the other end is mounted to a dual-axis goniometer, providing rotational motions around the *x*- and *y*-axes [Fig. 5(b)]. The goniometer is in turn mounted on a 3D stack-up of motorized linear stages (see Table SI), with each stage providing ±25 mm of travel range. The combination of the two-axis goniometer and 3D stage stack-up allows fine sample positioning so that the illuminated region of interest can be overlapped with the detection objective focal plane. The two-part design of the sample holder allows for the flexibility of designing different caddies to be best suited for specific types of specimens while still using the same dive bar to connect the caddy to the motion-control stages.

The flex-SPIM has two different modes of capturing volumetric information from a 3D sample: either by sample-scanning or by objective-scanning.

• Sample-scanning mode: the excitation light-sheet and detection objective remain stationary; the sample is moved via the *z*-stage of the 3D stage stack-up along the optical axis of the detection subsystem and images are sequentially collected. As the *z*-stage is already a necessary part of sample positioning, this approach is the simplest and most economical to implement. The motion range of *z*-stages is typically tens of mm; hence, sample-scanning enables volumetric imaging of large samples with depths up to the working distance of the detection objective (several mm or more). However, the imaging speed is limited by mechanical inertia of the stage and sample holder and by the communication overhead between the acquisition computer and the *z*-stage controller. Furthermore, the translational motion of the specimen can compromise normal biology.
• Objective-scanning mode: the movement of the detection objective piezo collar is synchronized with the second galvo mirror of each illumination arm—the light-sheet and piezo collar are scanned axially in concert (with a travel range of ±500 *µ*m set by the piezo collar) and images are sequentially collected (further details are described in Sec. II E). This approach, notwithstanding the additional cost of the piezo collar and its limited range of motion, enables fast volumetric imaging without moving the specimen and is preferred for our whole-brain functional imaging and simultaneous behavioral observation studies with zebrafish.

### E. Instrument control module

Each twin is independently controlled with a computer equipped with two Xeon E5-2650 v4 processors and 128 GB of 2400 MHz DDR4 RAM and seven PCIe slots, enough space for all the control cards. Instrument control and image acquisition are done through Micro-Manager.^29^ Custom software developed in LabVIEW (National Instruments) is used to independently control each of the 2D scanning galvo mirror systems and allows precise alignment (size and swept-rate control) of the excitation light-sheet relative to the sample (software available upon request). Collected images are written directly to a dual-disk array consisting of eight 7200 rpm, 4-TB disks.

**FIG. 6.**
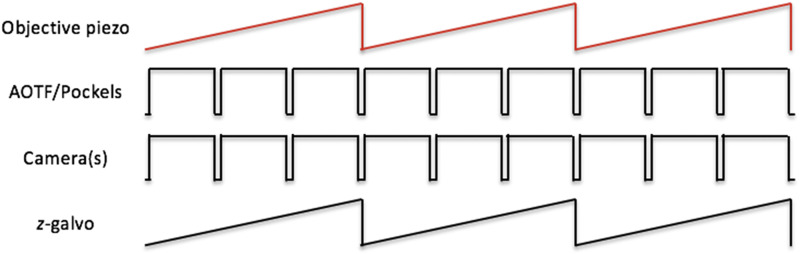
Schematic of control signal sequences for objective-scanning mode. The analog signal representing the position of the objective piezo collar is used as the master timing signal to generate control signals for the imaging cameras (both the fluorescence camera and behavior camera). The timing output of the fluorescence camera controls the AOTF/Pockels. The number of pulses driving the cameras, shown as 3 in the schematic here, determines the number of individual *z*-plane images to be recorded during a single *z*-scan cycle over the sample. The position signal of the objective piezo collar, appropriately scaled by a scaling amplifier, is also used to drive the *z*-galvo.

In the objective-scanning mode, the piezo collar’s controller serves as the master timing source. The analog position-readout of the piezo collar triggers a PicoScope, which is used to generate control signal sequences to synchronize the camera(s) with image capture. The position-readout of the piezo collar is also used to drive the position of the *z*-galvos. The waveform from the timing output of the scientific CMOS camera controls the AOTF and Pockels so that the sample is not illuminated during the camera readout. Analog control signals for the galvos and Pockels are appropriately conditioned by individual scaling amplifiers. A schematic of the control signal sequences is shown in (Fig. 6).

### F. Auxiliary module

Illumination can be selectively blocked with masks to avoid photosensitive regions or autofluorescent features in samples. For example, to avoid illuminating the zebrafish eyes while imaging neural activity, the excitation light is physically blocked with a pair of masks on each side of a horizontally positioned zebrafish and another mask for the front that covers both eyes [Fig. 5(a)]. These masks, fabricated out of black anodized aluminum, are mounted at the image planes of the illumination-scanning optics, each on 2D translational stages to permit their accurate positioning for different specimens or their complete removal from the illuminated field. A far-red light-emitting diode and a wide-field camera, positioned to view the sample from the bottom, enable view-finding and monitoring the tail behavior during neural imaging of the zebrafish (Fig. 5).

## III. INSTRUMENT PERFORMANCE

We characterized the 3D resolution of the flex-SPIM by measuring the point-spread function (PSF) with sub-diffraction fluorescent beads and then demonstrated the utility of the instrument for investigating biological systems by imaging the beating embryonic zebrafish heart, thick patient-derived tumor organoids, and neural activity in behaving zebrafish.

### A. Resolution characterization

**FIG. 7.**
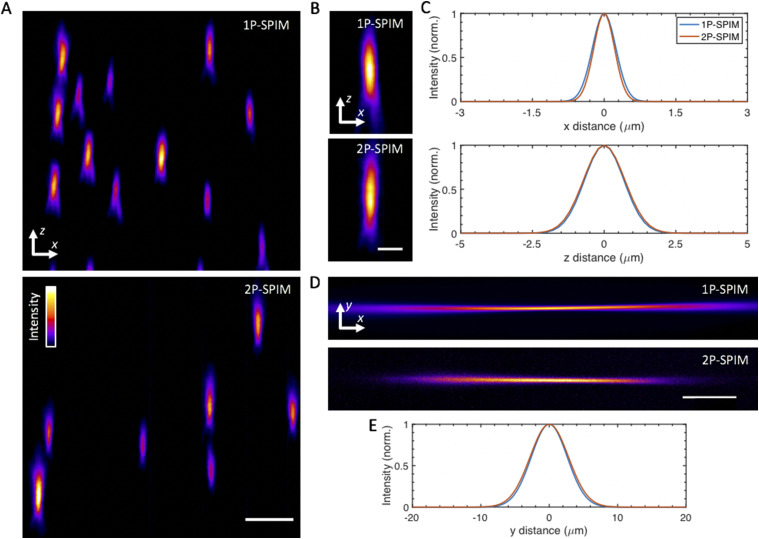
System imaging performance and characterization. (a) *y* maximum-intensity projections of agarose-embedded 175 nm fluorescent beads imaged at 44× magnification in 1P (top) and 2P excitation mode (bottom). A false-color (fire) lookup table was used to enhance visualization. (b) Selected *y* maximum-intensity projections of sub-diffraction fluorescent beads in 1P (top) and 2P mode (bottom). (c) Averaged lateral (top) and axial (bottom) full-width at half-maximum (FWHM) extents for the imaged beads, determined by Gaussian fits of 7 bead intensity profiles. The averaged lateral and axial FWHM ±SD values are 1P, 579 ± 15 nm and 1.67 *µ*m± 118 nm, respectively, and 2P, 528 ± 50 nm and 1.78 *µ*m ± 183 nm, respectively. Similar measurements at 11× magnification yield 1P, 1.2 *µ*m ± 86 nm and 1.73 *µ*m ± 387 nm and 2P, 1.17 *µ*m ± 232 nm and 1.81 *µ*m ± 370 nm for lateral and axial directions, respectively. (d) Experimental images of fluorescence excited by 1P (top) and 2P (bottom) Gaussian focused beams, which are scanned in the *y* direction to create virtual light-sheets. Images were acquired by illuminating a solution of rhodamine in the sample chamber. (e) Intensity line profiles for the focused beams in (d), taken at the center of focus, with approximate FWHM values: 1P, 6.2 *µ*m and 2P, 6.6 *µ*m. These FWHM values yield an averaged light-sheet thickness of ∼10 *µ*m across the 400 *µ*m extent along the *x* direction, centered around the Gaussian focus. Scale bars: (a) 5 *µ*m, (b) 2.5 *µ*m, and (d) 150 *µ*m.

We measured the system PSF with sub-diffraction (175 ± 5 nm diameter) fluorescent beads (PS-Speck Microscope Point Source Kit, P7220, Molecular Probes) embedded in 1.5% agarose, using the same detection objective (20×, NA = 1.0) with different tube lenses to yield two different magnifications of 44× and 11× (Table SI). Representative bead images and quantitative values of the PSF are presented in Fig. 7. On the whole, the instrument achieved ∼0.5 *µ*m and 1 *µ*m lateral resolution, for 44× and 11×, respectively, and ∼1.8 *µ*m axial resolution at both magnifications. This performance comes close to but does not achieve the full theoretical resolution limit determined by the NA of the detection objective, which is expected due to the practical compromises that we have made with the detection subsystem, as discussed previously in Sec. II C. Overall, the demonstrated resolution is quite suitable for a variety of imaging applications and represents the practical compromise between achieving a high resolution while maintaining a large field-of-view.

### B. Light-sheet imaging of the beating zebrafish heart

**FIG. 8.**
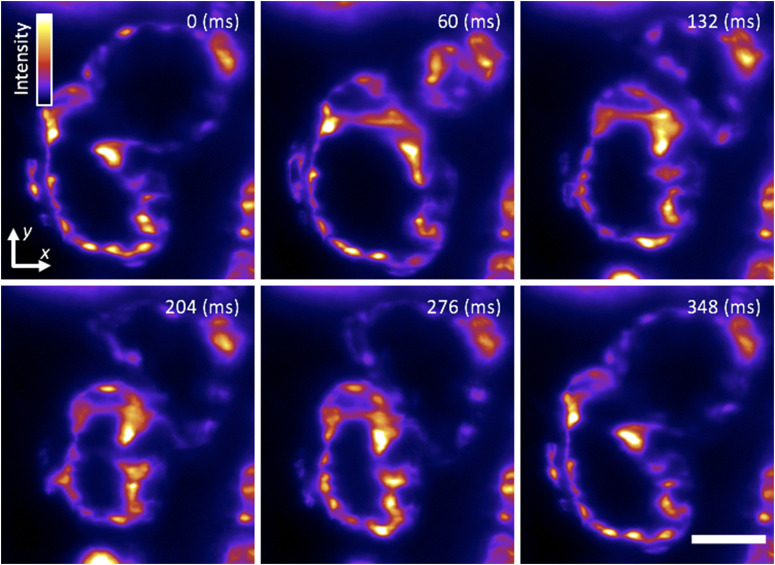
Cardiac light-sheet imaging. Single-plane SPIM recording of the beating heart in a live 5-dpf larval zebrafish with the endocardium fluorescently labeled (GFP), showing six distinct time points during the cardiac beating cycle. These subcellular 2D images are comparable to our previous efforts^21^ as well as recent work by others.^31^ A false-color (fire) lookup table was used to enhance visualization. Frames were captured with a magnification of 11× and 5 ms exposure time at a rate of 85 frames/s. Figure 9 (Multimedia view) shows a movie of the same data. Scale bar: 50 *µ*m.

**FIG. 9.**
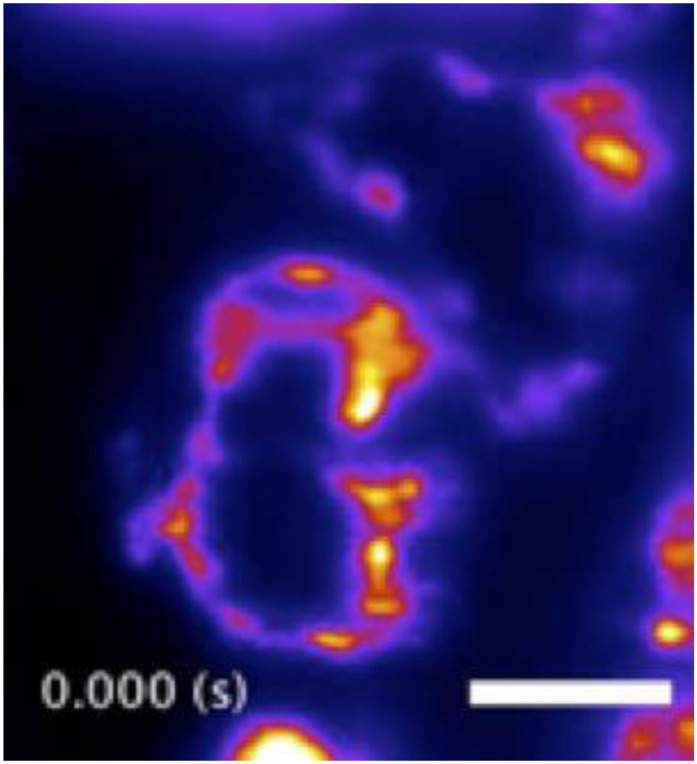
Light-sheet imaging of the dynamic motion of the beating heart of a 5-dpf transgenic larval zebrafish. Same dataset as presented in Fig. 8. Frames were captured at 85 Hz. Scale bar: 50 *µ*m. Multimedia view: https://doi.org/10.1063/1.5144487.110.1063/1.5144487.1

The vertebrate heart is a highly dynamic organ that starts to take its form and function early on during development.^32^ To gain insight into how the heart develops, studies of cells in their native dynamic and 3D context in the intact heart are needed. While the zebrafish is an ideal model system because of its optical and genetic accessibility,^33^ imaging is challenged by the heartbeat motion at 2–4 Hz and over tens of micrometers in amplitude.^34^ Retroactive synchronization techniques can align the 2D images by taking advantage of the quasi-periodicity of the heart motion.^21,31,35,36^ We acquired 2D images of the beating heart at a high-spatiotemporal resolution in a 5-dpf transgenic larval zebrafish with the vasculature fluorescently labeled. The 1P excitation (λ = 488 nm) light-sheet was parked in *z* to optically section through the beating heart of the agarose-embedded sample as we acquired images at 85 frames/s, 11× magnification, and subcellular resolution [Figs. 8 and 9 (Multimedia view)], showing that the flex-SPIM is fully compatible with existing retroactive synchronization techniques.^21,31,35,36^

### C. Imaging of thick, patient-derived tumor organoids

3D cell culture systems, such as spheroids or organoids, recapitulate the native physiology of multicellular tissues much better than 2D culture systems.^37^ Multicellular cancer organoids permit the study of disease development and patient-specific response to therapy.^38,39^ Unfortunately, such multicellular systems are scattering and aberrating, making them challenging to image with conventional instruments.

**FIG. 10.**
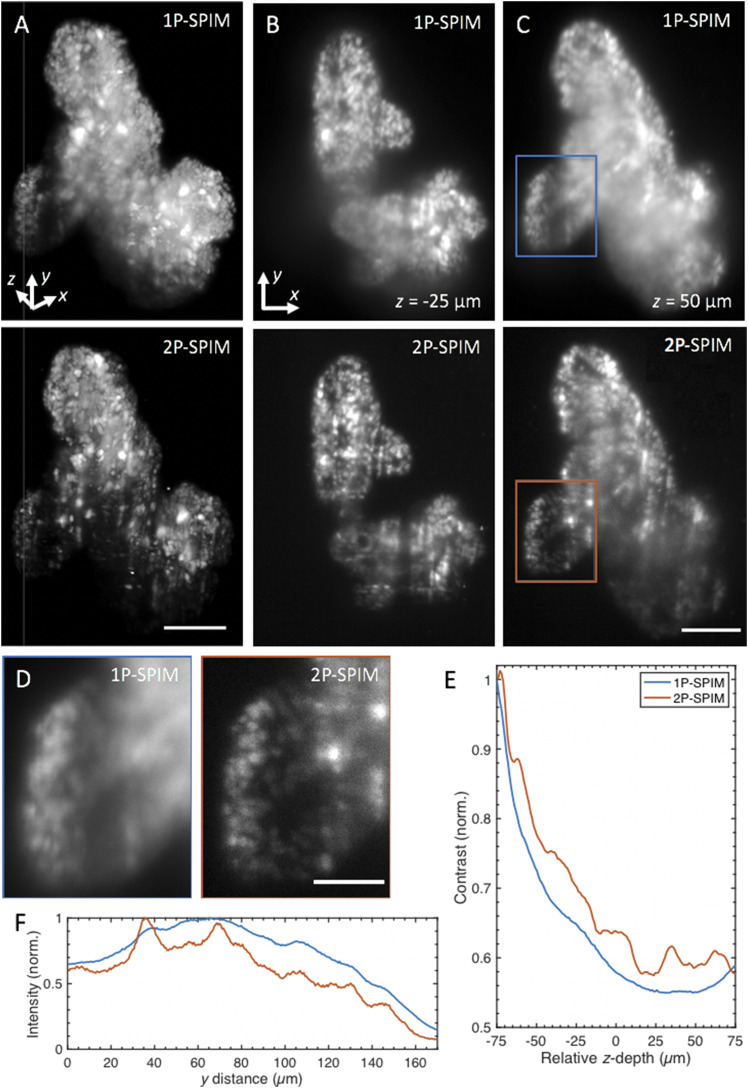
1P- and 2P-SPIM imaging of thick tumor organoids derived from a patient with colorectal cancer. (a) Volume rendering of fixed patient-derived tumor organoids expressing nuclear-localized H2B-GFP recorded in 1P (top) and 2P mode (bottom). Renderings show that the reduced background of 2P-SPIM enables better contrast throughout the imaged volume compared to 1P-SPIM. 3D organoid volume of ∼400 × 550 × 150 (*x*–*y*–*z*) *μ*m^3^ captured with a magnification of 11×, 1-*µ*m *z*-steps, and 150 ms exposure time. Figure 11 (Multimedia view) rotates the 3D-rendered volume of the same datasets. (b) and (c) are *x*–*y* image slices of (a) at *z* = −25 *µ*m (50 *µ*m from the surface) and *z* = 50 *µ*m (125 *µ*m from the surface), respectively. (d) Magnified images of the boxed regions in (c) for 1P (left) and 2P (right) mode revealing that 2P-SPIM resolves more cells than 1P-SPIM deep in the sample. (e) Quantification of image contrast as a function of *z*-depth. This plot shows quantitatively the improved contrast of 2P-SPIM over 1P-SPIM throughout the imaged volume in (a). Contrast calculated from the standard deviation of the pixel intensities from each *x*–*y* image slice and then normalized by the corresponding average image intensity. Each slice (from both modalities) is normalized against the surface slice (*z* = −75 *µ*m) of 1P-SPIM to show the degradation of performance as a function penetration depth. (f) Plot shows sum intensity along the *x* direction of images in (d) as a function of light-sheet propagation distance *y*. The longer NIR wavelength used in 2P-SPIM minimizes the scattering-induced degradation of the excitation light-sheet over longer propagation distances compared to the visible light used in 1P-SPIM. In both intensity profiles, intensity values were normalized by the global maximum. Scale bars: (a) and (c) 100 *µ*m and (d) 50 *µ*m.

**FIG. 11.**
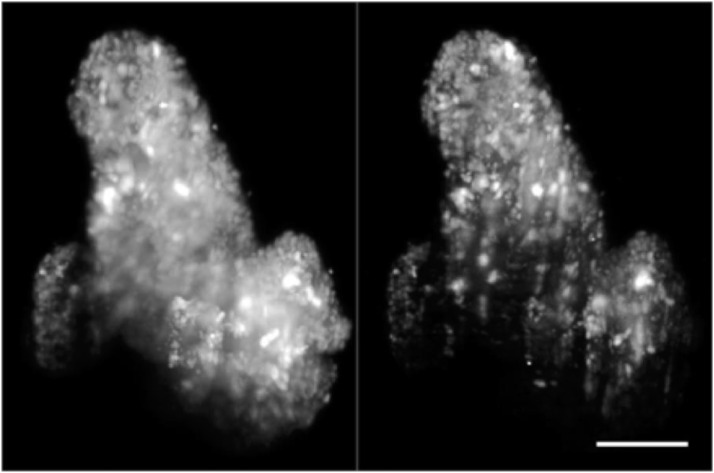
Volume rendering of fixed patient-derived tumor organoids expressing H2B-GFP, comparing images taken with one-photon (1P-, left) and two-photon excitation SPIM (2P-, right). Volumes are rotated around the *y* and *x* axes. Same datasets as presented in Fig. 10. Scale bar: 100 *µ*m. Multimedia view: https://doi.org/10.1063/1.5144487.210.1063/1.5144487.2

To show the advantages of the flex-SPIM for such opaque and optically heterogeneous samples, we imaged, at 11× magnification, chemically fixed, agarose-embedded organoids differentiated from cells derived from a colorectal cancer patient that had been engineered to transgenically express nuclear-localized H2B-GFP [Figs. 10(a) and 11 (Multimedia view)]. 2P-SPIM provides better contrast throughout the imaged volume because the reduced scattering at the longer wavelength (λ = 900 nm) enables better-preserved light-sheet shape over longer propagation distances compared to 1P [Figs. 10(b), 10(c), and 10(e)]. Even when the excitation light scatters, the fluorescence signal is still spatially restricted mainly to the central part of the light-sheet (where intensity is the highest) because of the quadratic dependence of the 2P-excited fluorescence signal on the excitation intensity. Thus, by mitigating the scattering-induced thickening of the light-sheet, 2P excitation with the flex-SPIM yields better effective 3D resolution than 1P (Fig. S2) and captures better images of labeled cells deep in the specimen [Figs. 10(d) and 10(f)].

### D. Whole-brain functional imaging of behaving zebrafish

SPIM enables recording of whole-brain neural activity in transgenic larval zebrafish.^15,16^ These implementations, however, potentially stimulate the photoreceptors and other photosensitive cells in the retina with the visible excitation wavelengths used during acquisition. Such illumination can reduce visual sensitivity to stimuli and interfere with visually driven processes.^20^ NIR (λ = 930 nm) 2P-SPIM overcomes this problem,^20^ achieving a recording depth of 120 *µ*m at a 1 Hz volume rate (sampled by nine *z*-planes).^17^

**FIG. 12.**
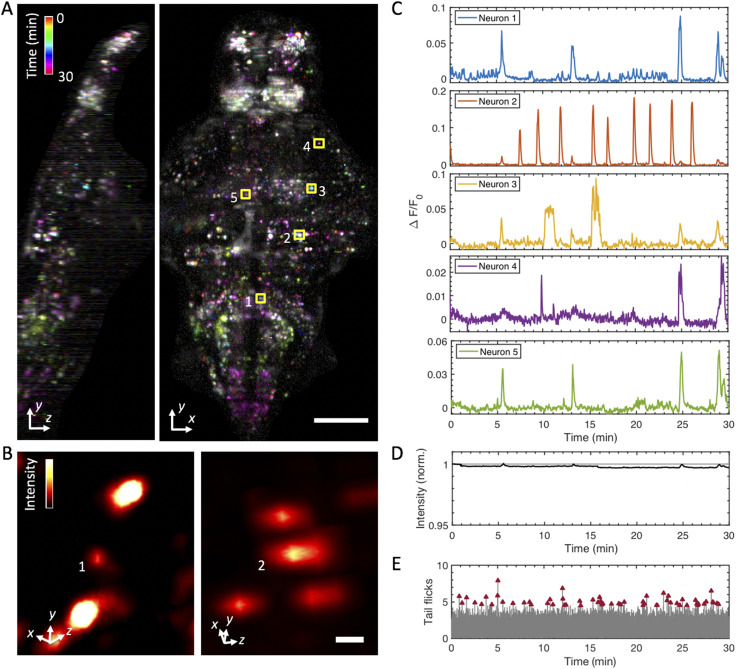
Whole-brain functional imaging at single-cell resolution in behaving 5-dpf transgenic larval zebrafish expressing nuclear-localized calcium indicator elavl3:H2B-GCaMP6s. (a) Maximum-intensity projections of calcium activity are color-coded in time over the 30-min recording window. Active neurons that exhibit fluorescence change during the recording appear as colored dots. Volume of 400 × 800 × 250 (*x*–*y*–*z*) *μ*m^3^ was sampled by 52 *z*-planes (4.8 *µ*m *z*-steps) at 0.5 Hz and 11× magnification. Figure 13 (Multimedia view) shows a 3D rendered movie of the same specimen. (b) Magnified volume renderings of neuron 1 (left) and neuron 2 (right) outlined in the *x*–*y* projection in (a), demonstrating cellular resolution in whole-brain 2P-SPIM imaging. The standard deviation projection along the temporal axis was used to generate the renderings, and a false-color (hot) lookup table was used to aid visualization. (c) Representative single-neuron activity traces extracted from the whole-brain recordings, calculated as ∆F/F from the manually selected neurons in (a). (d) Plot shows the total sum intensity of the entire imaged volume as a function of time, normalized to the average intensity of the first 5 min of acquisition (gray line at *y* = 1). Total intensity exhibits less than a 0.5% decrease in magnitude after the 30-min recording window, indicating that photobleaching is negligible. (e) Analysis of tail flick behavior as a function of time. Tail flicks (movements) obtained by taking the absolute difference in the pixel intensities from the tail region of the wide-field view of the sample, as shown in Fig. 5(b), normalized by the average image intensity corresponding to when there is no tail movement (gray lines). A threshold was applied to classify tail flicks and subsequently confirmed by manual inspection. Tail flicks (red arrowheads), indicating intended swimming behavior, occur throughout the recorded time window (average of ∼9 flicks ±2.2 every 5 min), thus suggesting minimal stress from the imaging conditions used. Scale bars: (a) 100 *µ*m and (b) 5 *µ*m.

**FIG. 13.**
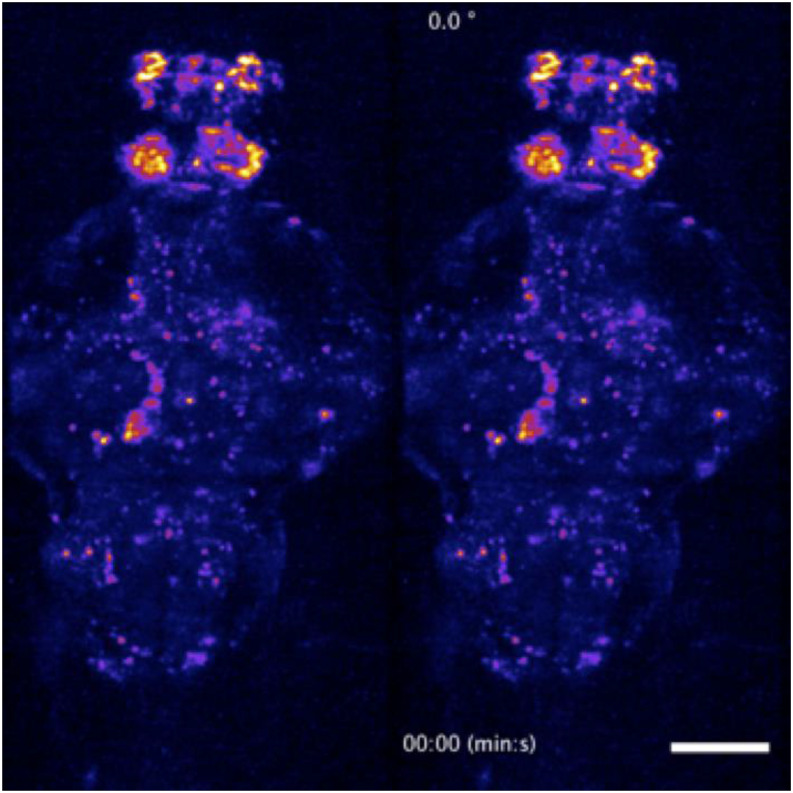
Dorsoventral (left) and rotating (right) maximum-intensity projections of a time-lapse recording of the whole-brain of the a 5-dpf transgenic larval zebrafish. Two-photon whole-brain functional light-sheet imaging was performed at a volumetric rate of 0.5 Hz. The video loops a 5-min recording as part of the data presented in Fig. 12. Scale bar: 100 *µ*m. Multimedia view: https://doi.org/10.1063/1.5144487.310.1063/1.5144487.3

We push the depth of 2P light-sheet functional imaging further with the flex-SPIM: more than doubling the volume size while maintaining high spatiotemporal performance [Figs. 12 and 13 (Multimedia view)]. By employing a trio of 2P excitation arms with masks to avoid direct laser illumination to the animal’s eyes, we imaged the entire (400 × 800 × 250 *µ*m^3^) brain of a 5-dpf zebrafish expressing a pan-neural calcium indicator (elavl3:H2B-GCaMP6s)^16^ at a 0.5 Hz volume rate (sampled by 52 *z*-planes) with single-neuron resolution and simultaneously monitored swimming behavior with a wide-field camera (Fig. 5). The temporal and spatial resolution of the time-series were sufficient to visualize individual active neurons across the brain [Figs. 12(a) and 12(b)] and capture single-neuron activity traces by calculating the relative fluorescence variation (∆F/F) as a function of time [Fig. 12(c)]. Imaging was carried out for 30 min continuously with 490 mW of total average laser power delivered to the sample from the three illumination arms. We observed no apparent signs of phototoxicity from the animal’s macroscopic behavior [Fig. 12(e)], cellular structure [Figs. 12(a) and 12(b)], or calcium dynamics [Fig. 12(c)]. Furthermore, the total fluorescence signal decreased by less than 0.5% after the 30-min, 900-*z*-stack acquisition [Fig. 12(d)], indicating that photobleaching was negligible. In future work, systematic analysis of the 4D functional imaging data acquired by the flex-SPIM, aided by state-of-the-art analysis pipelines such as CaImAn,^40^ will enable quantification of whole-brain single-cell activity patterns to gain insights into the neural basis of behavior.

## IV. DISCUSSION

We present the design and construction of the flex-SPIM, an instrument with two independently controlled light-sheet microscope-twins sharing the same multi-laser source, dramatically cutting the cost of the system. We demonstrate instrument versatility and application-specific customization by imaging a variety of specimens. In the same spirit as the OpenSPIM project,^41^ we offer a blueprint for optical developers to build and/or modify the flex-SPIM to serve user needs. Our design choice of upright (or inverted) detection, combined with our sample-mounting scheme using the caddy and the dive bar, allows for the flexibility in mounting different types of samples, from fixed tissues to live zebrafish embryos, but makes our instrument not easily amenable to multiview imaging,^42^ where the sample is imaged from multiple directions to improve axial resolution and coverage. On the other hand, a number of other modifications and enhancements could be implemented on the flex-SPIM to further optimize its performance for specific applications. Incoherent structured-illumination from intensity-modulated illumination patterns generated by the AOTF and/or Pockels cell would enhance contrast in more scattering specimens but would require additional exposures and post-processing.^43^ Confocal line detection using the rolling shutter of the sCMOS camera is an efficient alternative to structured-illumination and would allow rejection of non-ballistic photons.^44^ Designing a sample chamber rig with both temperature and CO_2_ control in conjunction with inverted detection would allow for optimized live imaging of cultured tissues as well as organoids.

Light-field microscopy could be readily deployed on the flex-SPIM, enabling high-contrast, synchronous volumetric imaging with SPIM-inspired selective-volume illumination.^45^ In addition, implementing multispectral imaging would improve signal multiplexing, either on the illumination path by rapid multispectral excitation^46^ or on the detection path by de-scanned detection via a confocal slit and diffraction grating.^47^ Further improvement is possible with our hyperspectral phasor software (HySP) for unmixing multiple spectrally overlapping fluorophores, even in the face of low signal-to-noise.^48^ The combination of HySP with a multispectral flex-SPIM design could thus enable dynamic visualization and quantitative analysis of many more important components and their interactions in intact specimens at high-resolution over extended durations.

## V. ETHICAL APPROVAL STATEMENT

All zebrafish raising and handling procedures followed guidelines established in the Guide for the Care and Use of Laboratory Animals by the University of Southern California, where the protocol was approved by the Institutional Animal Care and Use Committee (IACUC). All zebrafish lines used are available from ZIRC (zebrafish.org).

## SUPPLEMENTARY MATERIAL

See the supplementary material for a detailed list of the main flex-SPIM parts (Table SI), a panoramic photograph of the flex-SPIM (Fig. S1), optical transfer function comparisons of patient-derived tumor organoids (Fig. S2), and simulations of scan lens performance (Supplementary Note 1).

## References

[1] J. Vermot, S. Fraser, and M. Liebling, HFSP J. 2, 143 (2008). 10.2976/1.2907579 19404468 PMC2645566

[2] H. Siedentopf and R. Zsigmondy, Ann. Phys. 315, 1 (1902). 10.1002/andp.19023150102

[3] J. Huisken, J. Swoger, F. D. Bene, J. Wittbrodt, and E. Stelzer, Science 305, 1007 (2004). 10.1126/science.1100035 15310904

[4] L. Gao, L. Shao, B.-C. Chen, and E. Betzig, Nat. Protoc. 9, 1083 (2014). 10.1038/nprot.2014.087 24722406

[5] R. M. Power and J. Huisken, Nat. Methods 14, 360 (2017). 10.1038/nmeth.4224 28362435

[6] A. K. Glaser, N. P. Reder, Y. Chen, E. F. McCarty, C. Yin, L. Wei, Y. Wang, L. D. True, and Y. T. C. Liu, Nat. Biomed. Eng. 1, 0084 (2017). 10.1038/s41551-017-0084 29750130 PMC5940348

[7] T.-L. Liu, S. Upadhyayula, D. E. Milkie, V. Singh, K. Wang, I. A. Swinburne, K. R. Mosaliganti, Z. M. Collins, T. W. Hiscock, J. Shea, A. Q. Kohrman, T. N. Medwig, D. Dambournet, R. Forster, B. Cunniff, Y. Ruan, H. Yashiro, S. Scholpp, E. M. Meyerowitz, D. Hockemeyer, D. G. Drubin, B. L. Martin, D. Q. Matus, M. Koyama, S. G. Megason, T. Kirchhausen, and E. Betzig, Science 360, eaaq1392 (2018). 10.1126/science.aaq1392 29674564 PMC6040645

[8] M. Kumar, S. Kishore, J. Nasenbeny, D. L. McLean, and Y. Kozorovitskiy, Opt. Express 26, 13027 (2018). 10.1364/oe.26.013027 29801336 PMC6005676

[9] B. Yang, X. Chen, Y. Wang, S. Feng, V. Pessino, N. Stuurman, N. H. Cho, K. W. Cheng, S. J. Lord, L. Xu, D. Xie, R. D. Mullins, M. D. Leonetti, and B. Huang, Nat. Methods 16, 501 (2019). 10.1038/s41592-019-0401-3 31061492 PMC6557432

[10] V. Voleti, K. B. Patel, W. Li, C. Perez Campos, S. Bharadwaj, H. Yu, C. Ford, M. J. Casper, R. W. Yan, W. Liang, C. Wen, K. D. Kimura, K. L. Targoff, and E. M. C. Hillman, Nat. Methods 16, 1054 (2019). 10.1038/s41592-019-0579-4 31562489 PMC6885017

[11] R. Fiolka, Nat. Methods 16, 813 (2019). 10.1038/s41592-019-0542-4 31427758

[12] E. M. C. Hillman, V. Voleti, W. Li, and H. Yu, Annu. Rev. Neurosci. 42, 295 (2019). 10.1146/annurev-neuro-070918-050357 31283896 PMC6800245

[13] F. Cutrale, S. E. Fraser, and L. A. Trinh, Annu. Rev. Biomed. Data Sci. 2, 223 (2015). 10.1146/annurev-biodatasci-072018-021305

[14] K. McDole, L. Guignard, F. Amat, A. Berger, G. Malandain, L. A. Royer, S. C. Turaga, K. Branson, and P. J. Keller, Cell 175, 859 (2018). 10.1016/j.cell.2018.09.031 30318151

[15] M. B. Ahrens, M. B. Orger, D. N. Robson, J. M. Li, and P. J. Keller, Nat. Methods 10, 413 (2013). 10.1038/nmeth.2434 23524393

[16] N. Vladimirov, Y. Mu, T. Kawashima, D. V. Bennett, C.-T. Yang, L. L. Looger, P. J. Keller, J. Freeman, and M. B. Ahrens, Nat. Methods 11, 883 (2014). 10.1038/nmeth.3040 25068735

[17] S. Wolf, A. M. Dubreuil, T. Bertoni, U. L. Böhm, V. Bormuth, R. Candelier, S. Karpenk, D. G. Hildebrand, I. H. Bianco, R. Monasson, and G. Debrégeas, Nat. Commun. 8, 651 (2017). 10.1038/s41467-017-00310-3 28935857 PMC5608914

[18] P. J. Keller, A. D. Schmidt, J. Wittbrodt, and E. H. K. Stelzer, Science 322, 1065 (2008). 10.1126/science.1162493 18845710

[19] T. V. Truong, W. Supatto, D. S. Koos, J. M. Choi, and S. E. Fraser, Nat. Methods 8, 757 (2011). 10.1038/nmeth.1652 21765409

[20] S. Wolf, W. Supatto, G. Debrégeas, P. Mahou, S. Kruglik, J. M. Sintes, E. Beaurepaire, and R. Candelier, Nat. Methods 12, 379 (2015). 10.1038/nmeth.3371 25924070

[21] V. Trivedi, T. V. Truong, L. A. Trinh, D. B. Holland, M. Liebling, and S. E. Fraser, Biomed. Opt. Express 6, 2056 (2015). 10.1364/boe.6.002056 26114028 PMC4473743

[22] G. T. Reeves, N. Trisnadi, T. V. Truong, M. Nahmad, S. Katz, and A. Stathopoulos, Dev. Cell 22, 544 (2012). 10.1016/j.devcel.2011.12.007 22342544 PMC3469262

[23] D. V. Bower, N. Lansdale, S. Navarro, T. V. Truong, D. J. Bower, N. C. Featherstone, M. G. Connell, D. Al Alam, M. R. Frey, L. A. Trinh, G. E. Fernandez, D. Warburton, S. E. Fraser, D. Bennett, and E. C. Jesudason, Biol. Open 6, 1458 (2017). 10.1242/bio.026039 28821490 PMC5665464

[24] D. A. Lee, A. Andreev, T. V. Truong, A. Chen, A. J. Hill, G. Oikonomou, U. Pham, Y. K. Hong, S. Tran, L. Glass, V. Sapin, J. Engle, S. E. Fraser, and D. A. Prober, elife 6, e25727 (2017). 10.7554/elife.25727 29106375 PMC5705210

[25] J. Huisken and D. Stainier, Opt. Lett. 32, 2068 (2007). 10.1364/ol.32.002608 17767321

[26] B.-C. Chen, W. R. Legant, K. Wang, L. Shao, D. E. Milkie, E. Daniel, M. W. Davidson, W. Michael, C. Janetopoulos, X. S. Wu, J. A. Hammer, Z. Liu, B. P. English, Y. Mimori-Kiyosue, D. P. Romero, A. T. Ritter, J. Lippincott-Schwartz, L. FritzLaylin, R. D. Mullins, D. M. Mitchell, J. N. Bembenek, A. C. Reymann, R. Böhme, S. W. Grill, J. T. Wang, G. Seydoux, U. S. Tulu, D. P. Kiehart, and E. Betzig, Science 346, 1257998 (2014). 10.1126/science.1257998 25342811 PMC4336192

[27] M. Born and E. Wolf, Principles of Optics, 7th ed. (Cambridge University Press, 1999).

[28] E. A. Naumann, A. R. Kampff, D. A. Prober, A. F. Schier, and F. Engert, Nat. Commun. 13, 513 (2010). 10.1038/nn.2518 PMC2846983 20305645

[29] A. D. Edelstein, M. A. Tsuchida, N. Amodaj, H. Pinkard, R. D. Vale, and N. Stuurman, J. Biol. Methods 1, 10 (2015). 10.14440/jbm.2014.36 PMC4297649 25606571

[30] J. B. Pawley, Handbook of Confocal Microscopy, 3rd ed. (Springer US, 2006).

[31] M. Weber, N. Scherf, A. M. Meyer, D. Panáková, P. Kohl, and J. Huisken, elife 6, e28307 (2017). 10.7554/elife.28307 29286002 PMC5747520

[32] J. R. Hove, R. W. Köster, A. S. Forouhar, G. Acevedo-Bolton, S. E. Fraser, and M. Gharib, Nature 421, 172 (2003). 10.1038/nature01282 12520305

[33] D. Y. Stainer, Nat. Rev. Genet. 2, 39 (2001). 10.1038/35047564 11253067

[34] A. S. Forouhar, M. Liebling, A. Hickerson, A. Nasiraei-Moghaddam, H. J. Tsai, J. R. Hove, S. E. Fraser, M. E. Dickinson, and M. Gharib, Science 312, 751 (2006). 10.1126/science.1123775 16675702

[35] M. Liebling, A. S. Forouhar, M. Gharib, S. E. Fraser, and M. E. Dickinson, J. Biomed. Opt. 10, 054001 (2005). 10.1117/1.2061567 16292961

[36] J. M. Taylor, Front. Physiol. 5, 481 (2014). 10.3389/fphys.2014.00481 25566083 PMC4263077

[37] F. Strobl, A. Schmitz, and E. H. K. Stelzer, Nat. Protoc. 12, 1103 (2017). 10.1038/nprot.2017.028 28471459

[38] F. Pampaloni, B.-J. Chang, and E. H. K. Stelzer, Cell Tissue Res. 360, 129 (2015). 10.1007/s00441-015-2144-5 25743693

[39] T. Takahashi, Annu. Rev. Pharmacol. Toxicol. 59, 447 (2019). 10.1146/annurev-pharmtox-010818-021108 30113875

[40] A. Giovannucci, J. Friedrich, P. Gunn, J. Kalfon, B. L. Brown, S. A. Koay, J. Taxidis, F. Najafi, J. L. Gauthier, P. Zhou, B. S. Khakh, D. W. Tank, D. B. Chklovskii, and E. A. Pnevmatikakis, eLife 8, e38173 (2019). 10.7554/elife.38173 30652683 PMC6342523

[41] P. G. Pitrone, J. Schindelin, L. Stuyvenberg, S. Preibisch, M. Weber, K. W. Eliceiri, J. Huisken, and P. Tomancak, Nat. Methods 10, 598 (2013). 10.1038/nmeth.2507 23749304 PMC7450513

[42] R. Tomer, K. Khairy, F. Amat, and P. J. Keller, Nat. Methods 9, 755 (2012). 10.1038/nmeth.2062 22660741

[43] P. J. Keller, A. D. Schmidt, A. Santella, K. Khairy, Z. Bao, J. Wittbrodt, and E. H. K. Stelzer, Nat. Methods 7, 637 (2010). 10.1038/nmeth.1476 20601950 PMC4418465

[44] E. Baumgart and U. Kubitscheck, Opt. Express 20, 21805 (2012). 10.1364/oe.20.021805 23037300

[45] T. V. Truong, D. B. Holland, S. Madaan, A. Andreev, K. Keomanee-Dizon, J. Troll, D. E. Koo, M. McFall-Ngai, and S. E. Fraser, Commun. Biol. 3, 74 (2020). 10.1038/s42003-020-0787-6 32060411 PMC7021898

[46] A. M. Valm, S. Cohen, W. R. Legant, J. Melunis, U. Hershberg, E. Wait, A. R. Cohen, M. W. Davidson, E. Betzig, and J. Lippincott-Schwartz, Nature 546, 162 (2017). 10.1038/nature22369 28538724 PMC5536967

[47] W. Jahr, B. Schmid, C. Schmied, F. O. Fahrbach, and J. Huisken, Nat. Commun. 6, 7990 (2015). 10.1038/ncomms8990 26329685 PMC4569691

[48] F. Cutrale, V. Trivedi, L. A. Trinh, C.-L. Chiu, J. M. Choi, M. S. Artiga, and S. E. Fraser, Nat. Methods 14, 149 (2017). 10.1038/nmeth.4134 28068315

